# Improving antimicrobial prescribing in Irish primary care through electronic data collection and surveillance: a feasibility study

**DOI:** 10.1186/s12875-015-0280-3

**Published:** 2015-07-02

**Authors:** Sandra Galvin, Aoife Callan, Martin Cormican, Sinead Duane, Kathleen Bennett, Andrew W. Murphy, Akke Vellinga

**Affiliations:** Discipline of General Practice, School of Medicine, National University of Ireland Galway, Galway, Ireland; Discipline of Economics, J.E. Cairnes School of Business and Economics, National University of Ireland Galway, Galway, Ireland; Discipline of Bacteriology, School of Medicine, National University of Ireland Galway, Galway, Ireland; Department of Medical Microbiology, University Hospital Galway, Galway, Ireland; Department of Pharmacology & Therapeutics, Trinity College Dublin, Dublin, Ireland

**Keywords:** Antimicrobial prescribing, Quality of care, Primary care, Electronic data extraction

## Abstract

**Background:**

The increase in the spread of antimicrobial resistance (AMR) in bacterial pathogens and limited availability of new antimicrobials places immense pressure on general practitioners (GPs) to prescribe appropriately. Currently, electronic antimicrobial prescribing data is not routinely collected from GPs in Ireland for surveillance purposes to assess regional specific fluctuations or trends in antimicrobial prescribing. The current study aimed to address this issue by assessing the feasibility of remotely extracting antimicrobial prescribing data from primary care practices in Ireland, for the purpose of assessing prescribing quality using the European Surveillance of Antimicrobial Consumption (ESAC) drug specific quality indicators.

**Methods:**

Participating practices (n = 30) uploaded data to the Irish Primary Care Research Network (IPCRN). The IPCRN data extraction facility is integrated within the practice patient management software system and permitted the extraction of anonymised patient prescriptions for a one year period, from October 2012 to October 2013. The quality of antimicrobial prescribing was evaluated using the twelve ESAC drug specific quality indicators using the defined daily dose (DDD) per 1,000 inhabitants per day (DID) methodology. National and European prescribing surveillance data (based on total pharmacy sales) was obtained for a comparative analysis.

**Results:**

Antimicrobial prescriptions (n = 57,079) for 27,043 patients were obtained from the thirty study practices for a one year period. On average, study practices prescribed a greater proportion of quinolones (37 % increase), in summer compared with winter months, a variation which was not observed in national and European data. In comparison with national data, study practices prescribed higher proportions of β-lactamase-sensitive penicillins (4.98 % vs. 4.3 %) and a greater use of broad spectrum compared to narrow-spectrum antimicrobials (ratio = 9.98 vs. 6.26) was observed. Study practices exceeded the European mean for prescribing combinations of penicillins, including β-lactamase inhibitors.

**Conclusions:**

This research demonstrates the feasibility and potential use of direct data extraction of anonymised practice data directly through the patient management software system. The data extraction methods described can facilitate the provision of routinely collected data for sustained and inclusive surveillance of antimicrobial prescribing. These comparisons may initiate further improvements in antimicrobial prescribing practices by identifying potential areas for improvement.

## Background

Antimicrobial resistance (AMR) in bacterial pathogens represents one of the leading problems facing modern healthcare services globally. The development and spread of AMR results in a decline in the therapeutic effectiveness of antimicrobials and consequently a significant increase in patient morbidity and mortality [1]. The global increase of AMR can be attributed, in part, to over-use of antimicrobials in the primary care setting, where in Ireland and the UK approximately 80 % of antimicrobials used in human health care are prescribed [2, 3]. This problem is aggravated by a sustained decline in the development of new classes of antimicrobials over the last forty years [1, 4–6].

Surveillance of antimicrobial prescribing in Ireland is currently carried out annually by the Health Protection Surveillance Centre (www.hpsc.ie), which is part of the national healthcare provider, the Health Service Executive. Ireland is now ranked as the 10^th^ highest consumer of antimicrobials for systemic use in Europe and is classified as a mid-to-high consumer of antimicrobials when compared to other European countries [7]. While a reduction in antimicrobial use in the community was documented in 2008/2009, an increase has been reported in 2012 [7]. Significant inadequacies in the prudent use of antimicrobials in the Irish primary care setting have been identified both regionally and nationally [8, 9]. A high degree of prescribing variation within Ireland across counties has been reported for co-amoxiclav and ciprofloxacin and considerable seasonal fluctuations exist [7]. Even though national antimicrobial prescribing guidelines for common infections in primary care have been available since 2011, a recent study established that for urinary tract infections less than 40 % of prescriptions were in accordance with recommended first line therapy [8].

The European Surveillance of Antimicrobial Consumption (ESAC) Network established twelve quality indicators for antimicrobial prescribing. These indicators were developed by 22 recognised experts from 12 EU countries and were selected based on their relevance to reducing antimicrobial resistance, patient health benefit, cost effectiveness and public health policy makers [10]. The application of these indicators to European prescribing data has resulted in various publications [10–13]. These indicators can help identify both temporal and regional antimicrobial prescribing trends and encourage investigation and policy implementation [12]. Evaluation of total antimicrobial prescribing has allowed countries to benchmark their prescribing against other countries and make national recommendations where necessary [11].

One of the challenges for surveillance of antimicrobial resistance and antimicrobial consumption is to establish methods for exploitation of consistently collected data for surveillance purposes which also address sustainability and inclusiveness.

This research describes the evaluation of a process for application of the ESAC quality indicators to electronic prescribing data, obtained directly from primary care practices in Ireland for a one year period from Oct 2012 to Oct 2013. This data extraction process facilitated comparison with national and European data to identify potential areas for improved prescribing.

## Methods

Antimicrobial prescribing data for adult patients (≥18 years) from thirty selected primary care practices from the west of Ireland were used for the analysis. Selected practices were recruited to participate in a complex intervention (post data extraction) and eligibility and selection is described elsewhere [14].

The Irish Primary Care Research Network (IPCRN) is a network of primary care practices willing to participate in research through provision of anonymised practice data to directly enhance patient care (www.IPCRN.ie). Through participation in the IPCRN, the extraction of anonymised patient prescribing records is possible through the integration of an electronic tool for remote data extraction into the General Practitioner’s (GP) patient management software system. Anonymised practice data was automatically extracted from all study practices by a practice representative and uploaded to a secure server where data was anonymised and aggregated. Ethical approval for the study was obtained from the Irish College of General Practitioners (ICGP). Individual patient consent was not required as all data was anonymised. Patients were informed about their practices’ participation in the network through posters in the waiting room. Patients could request to be excluded from the study by informing their GP. Anonymised patient data was extracted for a one year period from mid-October 2012 to mid-October 2013. All aggregated practice data was pseudonymised and practice identity was not known to the researchers. Antimicrobial prescriptions were classified according to the Anatomical Therapeutic Chemical (ATC) classification system coding system and quantities of antimicrobials were expressed in DID (defined daily doses (DDD) per 1,000 patients per day) as previously described and explained in Table 1 [11]. Following the methods described by Adriaenssens *et al.* (2011), comparative quality assessment ranking of practice data was carried out using quartile distributions [11]. Comparative national and European sales data was obtained from the European Surveillance of Antimicrobial Consumption Network (ESAC-Net) database (accessed: September 2014) [15]. All analyses were conducted using Microsoft Office Excel (2007) and SPSS (version 20.0). Antimicrobial prescriptions were examined initially as counts and proportions of each ATC per practice. Given the nature of the data extraction, no missing observations were observed.

**Table 1:** ESAC drug-specific quality indicators for outpatient antibiotic use (Taken from Adriaenssens *et al*, 2011) [11]

Label	Description
J01_DID	consumption of antibacterials for systemic use (J01) expressed in DID
J01C_DID	consumption of penicillins (J01C) expressed in DID
J01D_DID	consumption of cephalosporins (J01D) expressed in DID
J01F_DID	consumption of macrolides, lincosamides and streptogramins (J01F) expressed in DID
J01M_DID	consumption of quinolones (J01M) expressed in DID
J01CE_%	consumption of β-lactamase-sensitive penicillins (J01CE) expressed as a percentage^a^
J01CR_%	consumption of combinations of penicillins, including β-lactamase inhibitors (J01CR) expressed as a percentage^a^
J01DD + DE_%	consumption of third- and fourth-generation cephalosporins [J01(DD + DE)] expressed as a percentage^a^
J01MA_%	consumption of fluoroquinolones (J01MA) expressed as percentage^a^
J01_B/N	ratio of the consumption of broad-{J01[CR + DC + DD + (F-FA01)]} to the consumption of narrow-spectrum penicillins, cephalosporins and macrolides [J01(CE + DB + FA01)]
J01_SV	seasonal variation of total antibiotic consumption (J01)^b^
J01M_SV	seasonal variation of quinolone consumption (J01M)^b^

^a^Percentage of total consumption of antibacterials for systemic use (J01) in DID

^b^Overuse in the winter quarters (October–December and January–March) compared with the summer quarters (July–September and April–June) of a 1 year period starting in July and ending the next calendar year in June, expressed as a percentage: [DDD (winter quarters)/DDD (summer quarters) −1] × 100

## Results

A total of 57,079 antimicrobial prescriptions (91 % acute, 9 % repeat) from 27,043 patients were obtained from the thirty participating practices (Table 2). The patient population receiving an antimicrobial was predominantly female (62.3 %) with the largest proportion of prescriptions in the 18 – 40 year age group. The proportion of antimicrobial prescriptions to patients receiving free health care accounted for 55 %.

**Table 2:** Patient variable characteristics (n = 27,043)

	% or mean (SD)	Median (range)
GMS^a^ status		
Public	54.5 %	
Private	45.5 %	
Gender		
Male	37.7 %	
Female	62.3 %	
Age		
Male	49 (19)	48 (18 – 107)
Female	47 (19)	43 (18 – 107)
All	48 (19)	45 (18 – 107)
18 - <40	40.5 %	
40 - <60	29.6 %	
60 - <80	22.8 %	
≥ 80	7 %	

^a^General Medical Services. Public patients who are entitled to free healthcare and in some cases, free medication

**Fig 1:**
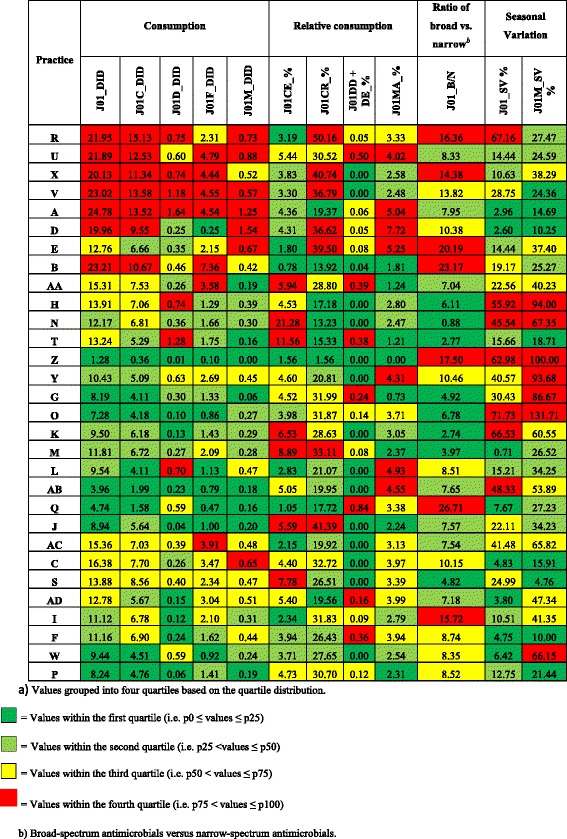
ESAC drug-specific quality indicators for antimicrobial use in 30 primary care practices^*a*^

**Fig. 2:**
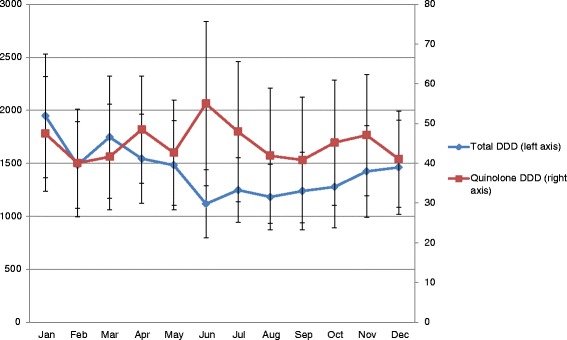
Total antimicrobial consumption (DDD) vs. quinolone consumption (DDD) by month

**Fig. 3:**
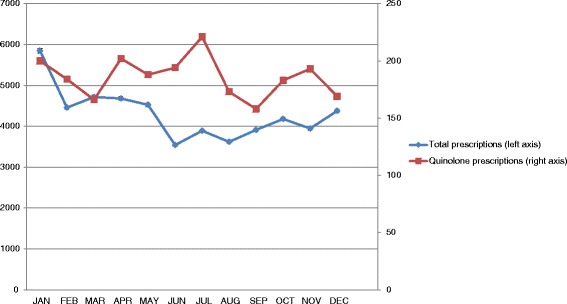
Total Number of antimicrobial prescriptions from study practices (n = 30) by month

**Fig. 4:**
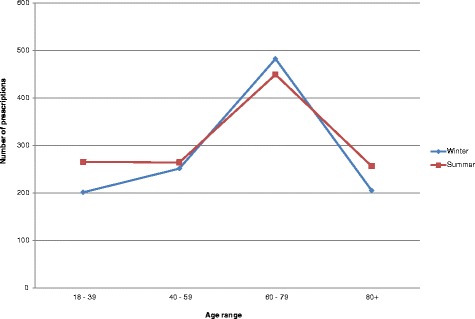
Number of quinolone prescriptions by age group and season

Figure 1 shows the indicator values for thirty practices grouped into four quartiles and ranked according to quartile distribution of the indicator values. Total prescribing of antimicrobials for systemic use ranged from 1.28 – 24.78 DID between practices. Of the four main antimicrobial indicator classes studied, the highest proportion of prescriptions were for penicillins (J01C - 53.4 %), followed by the macrolides, lincosamides and streptogramins (J01F – 17.5 %). Total prescribing was higher in winter months (Oct –Mar) in comparison with summer months (Apr – Sept) for 22/30 practices. In contrast, a higher proportion of quinolone prescriptions was observed in summer months, compared with winter months, for 20/30 practices with a mean increase of 34.7 %. The highest mean quinolone consumption (DDD) was observed in June (Fig. 2), while the highest mean number of quinolone prescriptions was observed in July (Fig. 3). An increase in the number of quinolone prescriptions (summer compared with winter) was observed for patients in the youngest age group studied (18 – 39 years), followed by patients in the oldest 80+ years category (Fig. 4). A decrease in the number of quinolone prescriptions (in summer compared with winter) was only noted for the 60-79 age group. In comparison with national prescribing data, study practices prescribed a higher proportion of β-lactamase-sensitive penicillins. An increase in the ratio of prescribing of broad-spectrum to narrow-spectrum antimicrobials was also observed for study practices (Table 3). Study practices exceeded the European average for the proportional use of combinations of penicillins; including β-lactamase inhibitors (J01CR) prescribed (Table 3). Seasonal variation of prescribing was substantially lower for study practices in comparison to both national and European averages.

**Table 3:** ESAC drug-specific quality indicators for antimicrobial use for study practices and national data^a^

Source	Consumption					Relative consumption				Broad vs. narrow	Seasonal variation	
	J01_DID	J01C_DID	J01D_DID	J01F_DID	J01M_DID	J01CE_%	J01CR_%	J01DD + DE_%	J01MA_%	J01_B/N	J01_SV %	J01M_SV %
Study Practices	13.21	7.05	0.46	2.31	0.44	4.98	26.85	0.12	3.18	9.98	25.85	44.80
National Data^a^	22.65	12.22	1.21	4.17	0.93	4.3	29.3	0.5	4.1	6.26	122.1	110.3
European Data^b^	20.98	10.26	2.14	3.11	1.71	6.19	21.99	2.05	7.86	29.34	127.65	111.35

^a^National and European Data (2011) obtained from http://ecdc.europa.eu/en/healthtopics/antimicrobial_resistance/esac-net-database/Pages/qualityindicators-primary-care.aspx

^b^Based on data available for Austria, Belgium, Bulgaria, Cyprus, Czech Republic, Denmark, Estonia, Finland, France, Germany, Greece, Hungary, Iceland, Ireland, Italy, Latvia, Lithuania, Luxembourg, Malta, Netherlands, Norway, Poland, Portugal, Romania, Slovakia, Slovenia, Spain, Sweden, United Kingdom

## Discussion

Traditionally, the quality of antimicrobial use in the primary care setting has been assessed from pharmacy sales data and is more reflective of antimicrobial consumption, rather than prescribing [7]. In Ireland this data can be obtained from a pharmaceutical market research company or the national Primary Care Reimbursement Service (PCRS) scheme. The PCRS database contains pharmacy dispensing data for “public” patients receiving free health care, which accounts for approximately 30 % of the population [7, 16]. This strategy does not take account potentially different prescribing practices in relation to “private”, fee paying patients. There is some evidence that private fee paying patients may be more likely to receive an antimicrobial in some settings [17]. Surveillance based on sales data also fails to take account of the prescribing / consumption difference associated with delayed prescribing, for which a prescription may be issued but may not be dispensed by the pharmacy. Delayed prescribing can result in a 25 % reduction in antimicrobial use [18].

The current study demonstrates the feasibility of extracting practice antimicrobial prescribing information of value in surveillance of resistance directly from a range of patient management software systems (*n* = 5) and more automatically. The methods described are similar to those routinely used in the UK through the Clinical Practice Research Datalink (CPRD) for which data is collected in the course of routine healthcare by general practitioners using coded classifications [19]. The use of anonymised practice data extraction allowed for passive patient consent with the provision of an opt-out system if needed. While not formally evaluated in the current study, cases of patients requesting to opt out of the study were not reported to the researchers. Such data extraction for other drugs and/or conditions and its application to research has the potential for far reaching benefits beyond the study of antimicrobial prescribing.

The application of the IPCRN to electronically extract and collect antimicrobial prescribing data directly form primary care practices has facilitated a tailored regional prescribing analysis as presented in this paper. The method of data collection presented is simple to use and provides a sustainable method of surveillance of antimicrobial prescribing, as it is fully integrated into all patient management software systems in Ireland. The availability of comparative data on antimicrobial prescribing can provide useful insights into the necessary steps required to improve antimicrobial prescribing in the primary care setting [20]. This has been successfully carried out on a European scale where benchmarking national data has resulted in an improvement stimulus in some countries [11]. Assessment of quality of prescribing for regional data allows for the generation of geographically personalised prescribing recommendations.

The current study has identified specific deviations in antimicrobial prescribing in the study region compared with national and European data. Whether these deviations are associated with sub-optimal care remains unclear and was not evaluated in the current study. However, greater prescribing of broad-spectrum compared to narrow-spectrum antimicrobials and seasonal variation of quinolone antimicrobials reported in study practices may be of particular concern, as both are identified as significant risk factors for *Clostridium difficile*–associated diarrhea (CDAD) and the development of antimicrobial resistance [21, 22]. The higher prescribing and consumption of quinolones in summer months compared with winter months was not evident from national data, therefore appears to be specific to the geographical region studied. Hospital based research in India identified increased prescribing during summer months and hypothesised that this is due to increased diagnostic and follow-up uncertainties [23]. The region studied in this research, experiences significant influx of tourists during the summer periods, making follow-up diagnostic testing for transient patients less feasible, which may explain a greater dependency on the reserve quinolone agents. However, the greatest increase in summer prescribing of quinolones was associated with patients in the 18-39 years age category, which may be associated with foreign travel.

While the presented analysis is informative, it should be noted that data extraction was not specifically carried out for analysis of antimicrobial prescribing and further patient based information may be useful to fully evaluate and understand prescribing quality. Additional disease-specific quality indicators have been developed which can allow for more informed patient specific analysis, for which the main prescribing indication is required [11, 24]. The proposed data extraction method may also exclude a significant proportion of antimicrobial prescribing from nursing homes, which are not always entered into the computer based system within the practice.

## Conclusions

Further routine utilisation and application of prescribing data obtained directly from primary care practices is needed to help create regional antimicrobial prescribing recommendations for GPs to optimise prescribing in primary care. Area specific prescribing data could also be used to provide further insights into regional antimicrobials demands based on population demographics. The IPCRN demonstrates the feasibility and acceptance of GPs in the extraction and utilisation of primary care data to improve patient care.

## Abbreviations

ESAC: European Surveillance of Antimicrobial Consumption
IPCRN: Irish Primary Care Research Network
DDD: Defined daily dose
DID: defined daily dose (DDD) per 1,000 inhabitants per day
AMR: Antimicrobial resistance
GP: General practitioner
ICGP: Irish College of General Practice
ATC: Anatomical Therapeutic Chemical classification system
PCRS: Primary Care Reimbursement Service
